# Supplementary material for: Effects of a land and aquatic exercise-based program on pain, mobility and quality of life in patients with chronic low back pain: a study protocol for a randomized controlled trial
Source: PLoS One. 2025 May 20;20(5):e0320858. doi: 10.1371/journal.pone.0320858 (PMC12091758; doi:10.1371/journal.pone.0320858)
Supplement: Attachment — Submitted filename: Response to Reviewers_Joana Borges.pdf [file pone.0320858.s003.pdf]

RESPONSE TO REVIEWERS

Dear Academic Editor Shabnam ShahAli,

Dear Reviewers,

We would like to express our gratitude for the opportunity to resubmit our manuscript to PLOS ONE. The feedback provided by the academic editor and reviewers was invaluable, and we have carefully addressed their suggestions and responded to their comments. We believe these revisions have significantly enhanced the quality of the manuscript.

Attached below are the reviewers' comments and a summary of the changes we made. Additionally, we have submitted an updated version of the manuscript with all revisions highlighted in yellow. Please feel free to contact us if you need any further information.

Journal requirements:

1. We have carefully reviewed and addressed all additional requirements and can confirm that our manuscript complies with PLOS ONE’s style guidelines, including the specified file naming requirements.

2. We confirm that this is a study protocol for a Clinical Trial. It was registered is ClinicalTrials.gov (https://clinicaltrials.gov/study/NCT06641570). This can also be confirmed in WHO database (https://trialsearch.who.int/Trial2.aspx?TrialID=NCT06641570). As the title page of the manuscript is lengthy, this information has been moved to page 2, before the Introduction section.

The complete date range for participant recruitment and follow-up was provided in the Methods section (page 8, lines 197-198 and page 8, lines 223-224).

3. We added a Funding Statement section in the manuscript. We also corrected this information and provided the correct grant numbers for the awards.

4. We included the statement “The funders had no role in study design, data collection and analysis, decision to publish, or preparation of the manuscript” in Funding Statement section (page 13, lines 427-431) and in the online form.

5. We removed the sentence form the Acknowledgments section and added this information to Funding Statement section (page 13, lines 420-423).

6. This information was wrongly provided. We added a Data Availability section in page 13, lines 424-426 to clarify it.

7. The Ethics section was replaced in the Methods section as asked (page 4, lines 113-128).

8. We rewrote the sentence of the Ethics section about confidentiality (page 4, lines 115-119).

9. We confirm that the reference list is complete and correct.

Review Comments to the Author

Reviewer #1: Good protocol paper with enough information. However there are some points needs to modify. Firstly, I did not satisfy with your work novelty. Are the outcome measures the only things that show your paper novelty?

Second, did you upload SPIRIT checklist to editor board with each item completed?

<u>Authors' response:</u> Thank you for your comment. We consider that the novelty is not only shown by the outcome measures. Some literature investigate the effects between land based programs and aquatic exercise programs, but we did not find publications that compared an aquatic exercise program to a combined exercise programs (aquatic and land based program), enlightened in page 3, lines 62-65. Additionally, the exercise cessation analysis may contribute to understand how the outcomes may modify after the patients end both exercise programs. This information was not very well clarified by literature and we also aim to investigate (page 3, lines 66-75).

The SPIRIT checklist was uploaded and completed with all the information that applies. A new version will be submitted with some corrections made.

Reviewer #2: I appreciate the opportunity to review this study. The authors have devoted significant time and effort to this study. I have provided some comments that should be addressed to improve the quality of the manuscript.

Introduction

1- Please add your specific hypotheses at the end of introduction.

Methods

2- The pre-specified outcome measures were not defined in terms of primary or secondary. Please define primary and secondary outcome measures in the manuscript.

3- The sample size should be calculated based on the pre-specified primary outcome. Did the authors consider the pain intensity (VAS) as the primary outcome in this study?

<u>Authors' response:</u> We appreciate your comment.

1 – We added the following information in page 3, lines 80-82 “We hypothesize that combined aquatic and land-based exercise decreases significantly pain, and functional disability, and increases quality of life in adults with CLBP”.

2 – Primary and secondary outcomes were defined in Outcomes subsection as suggested (page 9, line 242 and page 10, line 280).

3 – We confirm that VAS was used to calculate sample size as it a primary outcome (page 4, lines 103-108).

Reviewer #3: The manuscript has been accepted for publication following a comprehensive review. The reviewers found that it adheres to the journal's standards, with commendable methodology and data analysis.

Additionally, all required revisions have been properly addressed, enhancing the quality and clarity of the work.

Authors' response: Thank you very much for your comment.
