# Supplementary material for: Effects of a land and aquatic exercise-based program on pain, mobility and quality of life in patients with chronic low back pain: a study protocol for a randomized controlled trial
Source: PLoS One. 2025 May 20;20(5):e0320858. doi: 10.1371/journal.pone.0320858 (PMC12091758; doi:10.1371/journal.pone.0320858)
Supplement: Attachment — Submitted filename: Response_to_Reviewers_Joana_Borges_auresp_2.pdf [file pone.0320858.s004.pdf]

RESPONSE TO REVIEWERS

Dear Academic Editor Shabnam ShahAli,

Dear Reviewers,

We are grateful for the opportunity to resubmit our manuscript to PLOS ONE. The feedback from reviewers was helpful, and we have responded to their comments.

Attached below are the reviewers' comments along with a summary of the changes we made. Additionally, we have submitted an updated version of the manuscript with all revisions highlighted in yellow. Please feel free to reach out if you need any further information.

Journal requirements:

We confirm that the reference list has been thoroughly reviewed and is both complete and accurate.

Review Comments to the Author

Reviewer #1: Thanks for your good paper with enough information. The changes are acceptable. However, it is better to check out each item in spirit checklist. And it may make your paper more valuable if you add more outcome measures.

Authors' response: Thank you very much for your comment and contribution to our paper in order to enhance our work and make it more valuable. Regarding the suggestion to add more outcome measures, we have carefully considered this possibility. However, we believe that the outcomes already included are appropriate and sufficient to address the study objectives and provide relevant results.

The SPIRIT checklist was updated and we added information about the point 5d. We added the section “Author Contributions” to our manuscript in page 13, lines 432-446.

Reviewer #2: Dear authors,

Well done. My recommendations were addressed. My decision is now ACCEPTANCE.

Good Luck

Authors' response: Thank you very much for your comment and contributions.
