# Supplementary material for: Effects of a land and aquatic exercise-based program on pain, mobility and quality of life in patients with chronic low back pain: a study protocol for a randomized controlled trial
Source: PLoS One. 2025 May 20;20(5):e0320858. doi: 10.1371/journal.pone.0320858 (PMC12091758; doi:10.1371/journal.pone.0320858)
Supplement: Attachment — Submitted filename: Response_to_Reviewers_Joana_Borges_auresp_3.pdf [file pone.0320858.s005.pdf]

February 23, 2025

Dr. Shabnam ShahAli, Ph.D.,
Academic Editor
PLOS ONE

Manuscript ID:
PONE-D-25-01668R2

Manuscript title:
Effects of a land and aquatic exercise-based program on pain, mobility and quality of life in patients with chronic low back pain: A study protocol for a randomized controlled trial

Dear Editor,

My colleagues and I would like to thank you for the opportunity to resubmit our manuscript to *PLOS ONE*. We found that the academic reviewers' and editor comments were very helpful, and we have done our best to incorporate all their suggestions and reply to the comments. We believe that this has made a significant contribution to the overall quality of the manuscript.

Our responses are attached at the bottom of this letter. We have also submitted an updated version of our manuscript with all the changes highlighted in yellow. If you require any additional information, please do not hesitate to get in touch with us.

Thank you for considering our manuscript.

On behalf of all authors, yours sincerely,

Joana Borges

Additional Editor Comments:

The paper is generally well written and the concerns of the previous reviewers have been addressed and appear reasonable.

However, there are some concerns remaining from the statistical design and analysis perspective.

Authors' response: Thank you for your careful review and valuable suggestions for improving our paper. We have addressed your comments and revised the manuscript, highlighting the changes in yellow.

1. Please check other references to be sure that the effect size of 1.079 for the VAS being used for this study's sample size consideration is realistic. It does appear rather large and based on only one reference.

Authors' response: Thank you for your valuable comment. We acknowledge that the defined effect size is large; however, our choice is supported by the reference provided. In line with your suggestion, we have identified additional studies that report similar effect sizes:

- Effects of an aquatic exercise program on pain and quality of life in fibromyalgia patients; Cohen's *d* = 1,2 for pain reduction (VAS) after 12 weeks of intervention; Reference: Assis MR, Silva LE, Alves AM, Pessanha AP, Valim V, Feldman D, Neto TL, Natour J. A randomized controlled trial of deep water running: clinical effectiveness of aquatic exercise to treat fibromyalgia. *Arthritis Rheum*. 2006 Feb 15;55(1):57-65. doi: 10.1002/art.21693.
- Effectiveness of exercise therapy in adult non-specific acute, subacute and chronic low-back pain versus no treatment and other conservative treatments; Cohen's d = 1,1 for pain reduction (VAS) after 8 weeks of intervention; Reference: Hayden JA, van Tulder MW, Malmivaara A, Koes BW. Exercise therapy for treatment of non-specific low back pain. *Cochrane Database Syst Rev*. 2005 Jul 20;2005(3):CD000335. doi: 10.1002/14651858.CD000335.pub2.
- Impact of an exercise program on chronic pain and quality of life in the elderly; Cohen's d = 1,0; Reference: Geneen LJ, Moore RA, Clarke C, Martin D, Colvin LA, Smith BH. Physical activity and exercise for chronic pain in adults: an overview of Cochrane Reviews. *Cochrane Database Syst Rev*. 2017 Jan 14;1(1):CD011279. doi:

10.1002/14651858.CD011279.pub2. Update in: Cochrane Database Syst Rev. 2017 Apr 24;4:CD011279. doi: 10.1002/14651858.CD011279.pub3.

Action: We have incorporated these references into the manuscript. Please check page 4, line 105.

2. The inflation of 15% for the sample looks rather arbitrary. What is this based on? The sample size is small enough with the assumed large effect size and one does not want to come up short after such an effort.

Authors' response: We appreciate the editor's comment. In experimental studies, it is common practice to recruit approximately 15% more participants than the ideal sample size to ensure the robustness and validity of the results (Friedman et al., 2010). This approach accounts for potential participant dropouts, which may occur for various reasons unrelated to the intervention itself. Dropout rates in experimental studies typically range between 10% and 15% (Chow et al., 2017; Cramer et al., 2016) and compensating for these losses helps maintain the study's statistical power (Pedersen et al., 2017). By recruiting an additional 15%, we ensure that the final sample size remains sufficient for meaningful analysis.

Friedman LM, Furberg CD, DeMets DL. *Fundamentals of Clinical Trials*. Springer; 2010. https://doi.org/10.1007/978-1-4419-1586-3

Chow S-C, Shao J, Wang H, Lokhnygina Y. *Sample Size Calculations in Clinical Research* (3rd ed.). Chapman and Hall/CRC. 2017; doi: https://doi.org/10.1201/9781315183084

Cramer H, Haller H, Dobos G, Lauche R. A Systematic Review and Meta-Analysis Estimating the Expected Dropout Rates in Randomized Controlled Trials on Yoga Interventions. *Evid Based Complement Alternat Med.* 2016;2016:5859729. doi: 10.1155/2016/5859729.

Pedersen AB, Mikkelsen EM, Cronin-Fenton D, et al. Missing data and multiple imputation in clinical epidemiological research. Clin Epidemiol. 2017; 9:157-166. doi:10.2147/CLEP.S129785

3. There are three primary endpoints with an overall 0.05 significance level being used for the study. This involves multiple comparisons and thus needs an adjustment to the p-value or the type one error is inflated with multiple endpoints being considered. One may consider a Bonferroni type or other type of correction such as Holm-Bonferroni to the alpha level being used for each test. Likewise, there are three secondary endpoints and one could

challenge these results if a level of 0.05 is maintained. Ultimately, this could affect the actual sample size required for the entire study.

Authors' response: Thank you for your comment. We fully agree with the editor on the importance of controlling Type I errors when analyzing multiple outcomes. To address this, we will apply appropriate corrections, such as the Bonferroni adjustment, to minimize the risk of false positives (Ho et al., 2014).

Ho R. *Handbook of Univariate and Multivariate Data Analysis with IBM SPSS*. New York: CRC Press; 2014.

Action: The phrase: ”To control for Type I errors, multiple comparisons will be adjusted using the Bonferroni correction (i.e., alpha level divided by the number of tests) as suggested by several authors (e.g., Ho, 2014)” were added in page 11, lines 349-351.
