# Supplementary material for: Effects of a land and aquatic exercise-based program on pain, mobility and quality of life in patients with chronic low back pain: a study protocol for a randomized controlled trial
Source: PLoS One. 2025 May 20;20(5):e0320858. doi: 10.1371/journal.pone.0320858 (PMC12091758; doi:10.1371/journal.pone.0320858)
Supplement: Attachment — Submitted filename: Response to Reviewers_Joana Borges.docx [file pone.0320858.s002.docx]

Dear Academic Editor,

Regarding the indication that recruitment and data collection had already been completed, we confirm that there was a mistake. Although the initial plan was to complete the recruitment and data collection on the dates previously indicated in Clinical Trials, the process suffered delays due to logistical challenges. We already updated this information in the protocol registration with the new expected dates and they were accepted and corrected by Clinical Trials.

We apologize again for our oversight and discrepancy in the information provided and we hope that this situation could be analyzed again.

Best Regards,

Joana Borges and co-authors
